# Unveiling Lung Adenocarcinoma: Non-bacterial Thrombotic Endocarditis as the Debut Sign

**DOI:** 10.7759/cureus.45271

**Published:** 2023-09-14

**Authors:** Catarina R Rua, Mariana R Laranjeira, Ana C Dionisio, Maria A Mendes, Lourenco R Martins

**Affiliations:** 1 Rheumatology, Centro Hospitalar Vila Nova de Gaia/Espinho, Vila Nova de Gaia, PRT; 2 Internal Medicine, Centro Hospitalar Vila Nova de Gaia/Espinho, Vila Nova de Gaia, PRT; 3 Allergy and Immunology, Centro Hospitalar Vila Nova de Gaia/Espinho, Vila Nova de Gaia, PRT; 4 Radiology, Centro Hospitalar Vila Nova de Gaia/Espinho, Vila Nova de Gaia, PRT

**Keywords:** pleural effusion, multiple sclerosis, non-bacterial thrombotic endocarditis, pulmonary embolism, lung adenocarcinoma

## Abstract

Non-bacterial thrombotic endocarditis (NBTE) involves the deposition of fibrin and platelets on heart valves, frequently leading to systemic embolism. The association between NBTE and cancer demands thorough investigation in cases lacking an evident cause. This case report elucidates the clinical course of a nonsmoking woman in her sixties with NBTE linked to pulmonary adenocarcinoma. The patient, who had a history of multiple sclerosis (MS) and was receiving dimethyl fumarate treatment, presented to the emergency department with stroke-like symptoms. Diagnostic challenges arose due to preexisting motor sensory impairment from MS. Initial evaluations revealed hypocapnia and elevated inflammatory markers. Blood cultures were obtained twice, and imaging confirmed pneumonia, left pleural effusion, and chronic pulmonary embolism while excluding acute vascular events or intracranial hemorrhage. The first transthoracic echocardiogram (TTE) indicated no cardiac abnormalities. Treatment encompassed parenteral antibiotics, systemic anticoagulation, and admission to medical floors. Although the initial treatment yielded a positive clinical response, subsequent complications emerged. On the tenth day, the patient required additional interventions, including broad-spectrum antibiotics and supplemental oxygen. A follow-up chest X-ray revealed persistent pneumonia and pleural effusion, and blood cultures upon admission returned negative. A subsequent head MRI confirmed an embolic stroke and displayed evidence of MS progression. Around the twentieth day, empirical treatment for infective endocarditis was initiated, and an 8 mm vegetation on the aortic valve was identified via transesophageal echocardiography (TOE). Acute pulmonary edema prompted a transfer to the intermediate care unit. Further investigations, including left thoracocentesis and CT, unveiled exudate and metastatic lesions in the liver, ilium, and kidney. Unfortunately, on the twenty-fifth day, the patient experienced acute myocardial infarction, right leg ischemia, disseminated intravascular coagulation, and shock. Pleural fluid analysis revealed malignant cells suggestive of lung adenocarcinoma. This case underscores the pivotal role of timely NBTE recognition and the search for malignancy when workup for infective endocarditis and autoimmune panels is negative. Moreover, it emphasizes the significance of vigilant monitoring, particularly in immunocompromised individuals or those with preexisting neurological deficits, especially when new neurological symptoms manifest. These insights significantly contribute to the comprehension of NBTE management and its implications for analogous patient cohorts.

## Introduction

Non-bacterial thrombotic endocarditis (NBTE) is defined by the thickening of cardiac valves and/or the presence of vegetations resulting from non-infectious mechanisms and is susceptible to systemic embolization [1]. Cancer stands as a prominent instigator of NBTE and should be ruled out in NBTE instances lacking a definitive cause [2]. Malignancies most commonly linked to NBTE comprise mucin-secreting adenocarcinomas affecting the lung, ovary, biliary system, pancreas, breast, and stomach [2,3]. The physiopathology of NBTE is still inadequately comprehended [4]. Clinical presentation varies from asymptomatic to systemic embolism, particularly to the brain, spleen, kidney, or digits [4]. Cardiac and cerebral embolization, when present, are symptomatic [4]. The investigation process entails distinguishing NBTE from infective endocarditis, elucidating the primarily associated condition, and evaluating valve abnormalities using echocardiography [2,4,5]. Treatment of this condition includes systemic anticoagulation control of the neoplasm and, rarely, cardiac surgery [2,4,5]. Usually, the underlying malignancy is often advanced with poor prognosis and palliative treatment may be most appropriate [5]. We report an intriguing case of NBTE presenting as an embolic stroke in the context of lung adenocarcinoma.

## Case presentation

**Figure 1:**
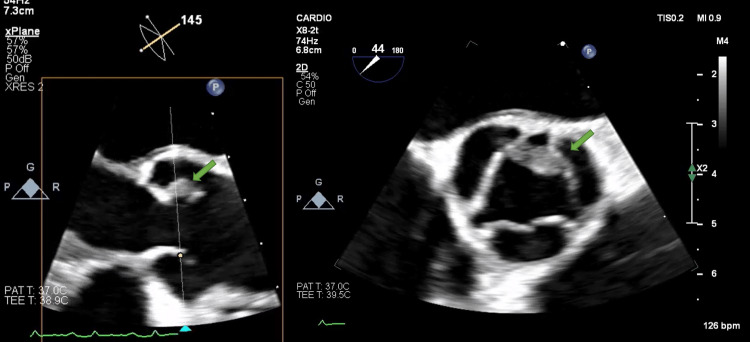
Biplanar TOE showing an 8 mm echogenic mass between the coronary and noncoronary cusps of the aortic valve (indicated by the green arrows) TOE: transesophageal echocardiography

We report the case of a nonsmoker in her sixties with NTBE secondary to pulmonary adenocarcinoma. Relevant past medical history included MS diagnosed a decade ago and had been undergoing treatment with dimethyl fumarate 240 mg twice daily since 2017. Additionally, she had a major depressive disorder diagnosed three years before her hospital admission. This was accompanied by a challenging mental health history, including multiple suicide attempts. Her psychiatric regimen involved olanzapine, venlafaxine, and lorazepam. Regular appointments with a psychiatrist were scheduled due to her condition and a history of benzodiazepine abuse. Upon presenting at the emergency department, the patient reported several concerning symptoms. These included a drooping left facial lip, slurred speech, and delirium. Further evaluation revealed her medical records indicating a baseline crural paresis grade 4 on her right leg and generalized hyperreflexia with clonus, which had persisted for more than 10 seconds. In the emergency room (ER), her speech exhibited notable impairment. It was characterized by slurred speech, dysphemia, paraphasia, and errors in repetition. Importantly, a recent ER admission had been necessitated by benzodiazepine abuse, with a positive test for lorazepam on a urinary drug panel (although excessive intake couldn't be ruled out). During the physical examination, additional neurological signs emerged such as left grasp, left glabellar, and palmomental reflexes, pronation of the right upper extremity, a dystonic posture of the right foot, mild spasticity of the lower extremities, and motor deficits in the right lower extremity. The patient denied experiencing fever, cough, shortness of breath, or any other cardiorespiratory symptoms. Vaccination status was up to date. Notably, prior screenings revealed normal colorectal results five years ago, with pending cervical and breast screenings. However, due to her lack of smoking history, lung cancer screening had not been performed. Initial observations included diminished lung sounds on the left hemithorax and regular tachycardia upon cardiopulmonary auscultation. Remarkably, no digital clubbing or palpable lymphadenopathy was present. Subsequent workup tests unveiled arterial blood gas analysis with hypocapnia, sinus tachycardia, and a blood count suggesting leukocytosis with neutrophilia. Her C-reactive protein (CRP) level was measured at 50 mg/L. Two sets of blood cultures were collected for further investigation. Chest radiography demonstrated bilateral pneumonia with a left pleural effusion while an electrocardiogram indicated sinus tachycardia. A head CT scan ruled out acute vascular events or bleeding. Further imaging, including a subsequent chest CT, revealed a moderate left pleural effusion, consolidation of the left lower lobe, and opacities/ground-glass changes in the left lung, indicative of infectious/inflammatory alterations and chronic pulmonary embolism. Small adenopathies in the mediastinum and hilum were also observed, suggesting a reactive etiology. No abdominal changes were reported. transthoracic echocardiogram (TTE) results showed no cardiac abnormalities. Treatment encompassed parenteral antibiotics, systemic anticoagulation, and admission to medical floors. Physical rehabilitation and speech therapy were initiated to address neurological deficits, resulting in an initial positive clinical response. However, subsequent complications emerged. On the tenth day, the patient's condition required further interventions, which involved administering broad-spectrum antibiotics and providing supplemental oxygen. Subsequently, a follow-up chest X-ray revealed left-sided pneumonia, albeit with a reduced pleural effusion. It's important to note that blood cultures returned negative results, and this was also the case for atypical agents serology and autoimmune panel. A head MRI demonstrated diffusion restriction in the cortical and subcortical gray matter in the left frontoparietal and temporal regions, as well as the right occipital region. These findings suggested embolism and indicated evidence of MS progression. Around the twentieth day of hospitalization, empirical treatment for infective endocarditis was initiated. Transesophageal echocardiography (TOE) revealed an 8 mm vegetation on the aortic valve, despite the patient having a minimal likelihood of infective endocarditis based on the modified Duke criteria. Despite her condition, with fever and hypoxemia persisting and no improvement observed despite two different antibiotic courses, a new set of blood cultures was obtained, and empiric therapy for infective endocarditis was initiated. A TOE uncovered an 8 mm vegetation situated between the coronary and noncoronary aortic cusps (Figure 1), providing crucial insights into the patient's condition.

**Figure 2:**
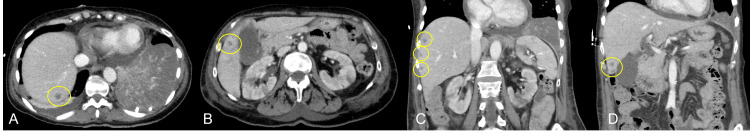
Abdominal CT showing multiple hypodense liver lesions with peripheral ring enhancement (yellow circles), raising suspicion for metastatic lesions

**Figure 3:**
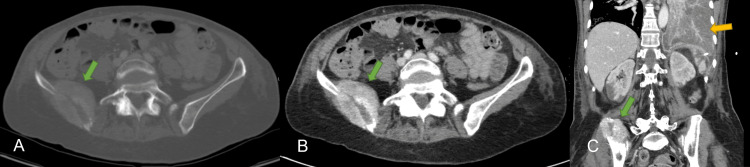
CT of the pelvis showing a right iliac bone mass with a soft tissue component (green arrows), highly suggestive of a metastatic lesion Note the consolidation of the entire left lower lobe (yellow arrow).

**Figure 4:**
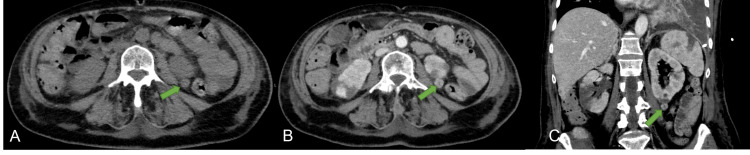
Pre-contrast CT (A) and post-contrast CT (B and C) showing a solid nodule located in the inferior pole of the left kidney (arrows) with contrast enhancement, highly suspicious of a renal neoplasm

She was later transferred to the intermediate care unit because of acute pulmonary edema. There, the left pleural effusion was drained and sent for analysis. In addition, a new CT of the thorax/abdomen/pelvis showed evidence of metastatic lesions in the right liver lobe (Figure 2), right ilium (Figure 3), and left kidney (Figure 4).

Regrettably, on the twenty-fifth day, the patient experienced acute myocardial infarction, right leg ischemia, disseminated intravascular coagulation, and shock. Later, cytology and histochemistry of pleural fluid revealed evidence of lung adenocarcinoma. No autopsy was required by the family members.

## Discussion

NTBE is found in approximately 1.0% of all autopsies, most commonly occurring from the fourth through eighth decades of life and equally affecting males and females [1]. In the biggest study of culture-negative endocarditis, NBTE accounted for 2.5% of cases [4]. The physiopathology of NTBE may include the impairment of the endothelium induced by circulating substances like tumor necrosis factor or interleukin-1, which may activate the coagulation process, leading to the accumulation of platelets and fibrin, especially when the coagulation system is already activated [4]. In the presence of negative blood cultures, negative atypical agents serology, and negative autoimmune panel, NBTE should be suspected [3,4]. Imaging should be performed as soon as possible and since TEE could be normal, a TOE should be performed if clinical suspicion remains high [3,4,6]. Typical findings on TOE include usually small vegetations (<1 cm), wide-based, and irregular in contour [4]. We presented a case of an immunosuppressed patient in whom one of the earlier signs of metastatic lung adenocarcinoma was NTBE. Unfortunately, the cancer diagnosis was not made promptly, delaying appropriate treatment. An important takeaway from this case is the value of pursuing a follow-up chest CT when clinical progression falls short of anticipated results, even when initial chest X-ray findings are inconclusive. Additionally, considering an earlier pleural fluid analysis could have contributed to an earlier diagnosis. NBTE diagnosis needs a high level of clinical suspicion, corresponds to only 1-3% of all types of endocarditis, and post-mortem studies suggest that 75% of cases are cancer-related [5]. According to Rahouma et al., lung cancer is the most common malignancy associated with NTBE, and the latter leads to an inaugural cancer diagnosis in up to 66.4% of cases [5]. Lung cancer exhibits a diverse range of clinical and radiological manifestations and can mimic both infectious and inflammatory diseases of the lung accompanied by an unpredictable course of progression and varying natural history [7]. Consequently, timely identification and early recognition are imperative. Cancer risk in patients with MS is contradictory; some studies report an increase in frequency compared with the general population [8]. Limited information exists regarding the potential occurrence of malignancies in individuals with MS who undergo treatment with dimethyl fumarate [8]. In the case presented, NTBE was only the tip of the iceberg and brings to light the need for closer surveillance of cancer (especially breast, colon, and cervical cancer) in MS patients who are under immunosuppression drugs. This also opens up the discussion of whether we need to include screening for lung cancer in non-smoking individuals. In addition, in a patient with pre-existing neurological deficits, we should not devalue new complaints, even with a prior history of prescription drug abuse. Finally, our patient presented pneumonia that was unresponsive to two courses of antibiotics, which should raise a suspicion of atypical presentation of lung cancer, and further studies should be performed.

## Conclusions

NTBE is a challenging diagnosis that can lead to disastrous consequences if left undiagnosed. It should be considered in patients with embolic complications and no evident cause. Underlying neoplastic disease should be suspected, especially if the patient is under immunosuppressive drugs.
